# Genome-Wide Association Study and Transcriptome Differential Expression Analysis of the Feather Rate in Shouguang Chickens

**DOI:** 10.3389/fgene.2020.613078

**Published:** 2020-12-22

**Authors:** Xiayi Liu, Zhou Wu, Junying Li, Haigang Bao, Changxin Wu

**Affiliations:** ^1^National Engineering Laboratory for Animal Breeding, Beijing Key Laboratory of Animal Genetic Improvement, College of Animal Science and Technology, China Agricultural University, Beijing, China; ^2^Animal Breeding and Genomics, Wageningen University & Research, Wageningen, Netherlands

**Keywords:** Shouguang chicken, early-feathering, late-feathering, genome-wide association study, RNA sequencing, *SPEF2*, *PRLR*

## Abstract

The feather rate phenotype in chicks, including early-feathering and late-feathering phenotypes, are widely used as a sexing system in the poultry industry. The objective of this study was to obtain candidate genes associated with the feather rate in Shouguang chickens. In the present study, we collected 56 blood samples and 12 hair follicle samples of flight feathers from female Shouguang chickens. Then we identified the chromosome region associated with the feather rate by genome-wide association analysis (GWAS). We also performed RNA sequencing and analyzed differentially expressed genes between the early-feathering and late-feathering phenotypes using HISAT2, StringTie, and DESeq2. We identified a genomic region of 10.0–13.0 Mb of chromosome Z, which is statistically associated with the feather rate of Shouguang chickens at one-day old. After RNA sequencing analysis, 342 differentially expressed known genes between the early-feathering (EF) and late-feathering (LF) phenotypes were screened out, which were involved in epithelial cell differentiation, intermediate filament organization, protein serine kinase activity, peptidyl-serine phosphorylation, retinoic acid binding, and so on. The sperm flagellar 2 gene (*SPEF2*) and prolactin receptor (*PRLR*) gene were the only two overlapping genes between the results of GWAS and differential expression analysis, which implies that *SPEF2* and *PRLR* are possible candidate genes for the formation of the chicken feathering phenotype in the present study. Our findings help to elucidate the molecular mechanism of the feather rate in chicks.

## Introduction

The feather rate phenotype in newborn chicks can be observed within 24 h after hatching, based on the relative length of the primary feathers and primary-covert feathers. A chick with its primary feathers longer than its primary-covert feathers by more than 2 mm is caused by the early-feathering (EF) phenotype; otherwise, it would be caused by the late-feathering (LF) phenotype. The feather rate phenotype is sex-linked and controlled by the K/k+ allele. The K allele is dominant and contributes to the LF phenotype, relatively the k+ allele is recessive and promotes the EF phenotype (Warren, 1925). The autosexing system of the feather rate phenotype is widely used in the chicken industry because of its simple operation, low price, and the low stress response of chicks. Many chicken genes associated with economic traits have been continuously discovered and studied (Li et al., 2019, 2020). The relationship between the feather rate phenotype and production performances were also investigated. Durmus et al. (2010) reported that the EF group of brown layers had higher hen-day egg production and live weight at sexual maturity in comparison with those of the LF group. Goger et al. (2017) observed that the EF groups generally had higher egg production and hatching performances except for egg weight. Compared to yellow-feathered broilers with the EF phenotype, yellow-feathered broilers with the LF phenotype had significantly higher breast muscle weight, breast muscle rate, abdominal fat weight, and abdominal fat rate (Hu et al., 1995).

It was reported that when the endogenous retrovirus ev21 was inserted into chromosome Z, it was closely linked to the K allele in the White Leghorn, which may increase the infection of leukemia virus in LF chickens (Bacon et al., 1988). However, the provirus ev21 was found in both EF and LF feathering Rhode Island Red commercial layers (Boulliou et al., 1992) and no ev21 insertion was detected in LF feathering chickens of some chicken breeds (Smith and Fadly, 1988; Takenouchi et al., 2018), which demonstrated that endogenous virus ev21 insertion was not the causative mutation of the feather rate phenotype. A detailed molecular analysis in LF chickens revealed the presence of a tandem duplication of 176,324 bp of the partially duplicated prolactin receptor (*dPRLR*) and sperm flagellar 2 genes (*dSPEF2*) which could be a candidate gene for the LF phenotype (Elferink et al., 2008; Bu et al., 2013). The *dPRLR* encodes a membrane-spanning receptor identical to the original *PRLR* except for a deletion of a 149-amino acid C-terminal tail (Bu et al., 2013). It can be activated by prolactin and functionally coupled to the JAK-STAT5 signaling pathway (Bu et al., 2013, 2015). Li (2012) and Huang (2014) found that many EF feathering chicks had the tandem duplication in Chinese native chicken breeds, which suggested that *dPRLR* was not the determinant of the LF feathering phenotype. Luo et al. (2012) found that the expression level of the prolactin receptor (*PRLR*) gene in LF chickens was 1.78 times higher than that in EF chickens. However, Zhao et al. (2016) reported that there was no significant difference between the *PRLR* expression levels of the EF and LF chicks in their study. Because there was an extremely significant difference in the expression level of *SPEF2*, but not of *PRLR* between the EF and LF chickens, Zhao et al. (2016) suggested that *SPEF2* should be a good candidate gene for the chicken feather rate phenotype rather than *PRLR*. In summary, it seems that the genetic determinant of the EF and LF phenotypes is still debatable.

In the present study, a total of 56 Shouguang chicks were genotyped with a 600K SNP Axiom^®^ Genome-Wide Chicken Genotyping Array. The genome-wide association study (GWAS) was performed to map candidate genomic regions related to the feather rate; RNA sequencing data from the hair follicle tissue of the EF and LF samples were analyzed and many differentially expressed genes (DEGs) were checked. *SPEF2* and *PRLR* were screened by both GWAS and RNA sequencing analysis, and therefore could be regarded as two reliable candidate genes for the LF phenotype. This work paved the way for further studies to explain the molecular biological process of the formation of the EF and LF phenotypes in the future.

## Materials and Methods

### Ethics Statement

All experimental procedures and used animals were approved by the Ethics Review Committee for Laboratory Animal Welfare and Animal Experiment of China Agricultural University (Approval number: AW28090202-1).

### Genome-Wide Association Study

**FIGURE 1:**
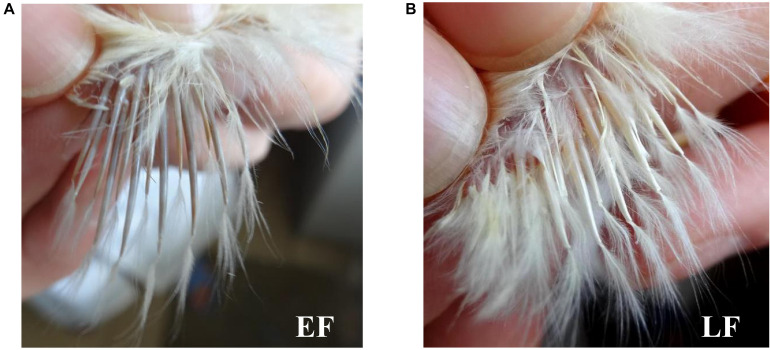
Chicken feather rate phenotypes. **(A)** Chicken early-feathering phenotype. **(B)** Chicken late-feathering phenotype. One-day-old chicks with primary feathers longer than primary-covert feathers by more than 2 mm were identified with the early-feathering phenotype, otherwise, they would be identified with the late-feathering phenotype.

Phenotypes of the feather rate in one-day-old chicks were distinguished according to Xie et al. (1985). Briefly, one-day-old chicks with primary feathers longer than their primary-covert feathers by more than 2 mm were confirmed to have the EF phenotype, otherwise, they were confirmed to have the LF phenotype (Figure 1). Fifty-six female Shouguang chicken blood samples, including 28 EF samples and 28 LF samples, were collected from the Experimental Chicken Farm of China Agricultural University. Total DNA of each sample was extracted from chicken blood using the TIANamp 98 Blood DNA Kit (Cat. DP348, TIANGEN, Beijing, China) according to the supplied protocol. Genotyping was performed by a commercial company using a 600K SNP Axiom^®^ Genome-Wide Chicken Genotyping Array (Kranis et al., 2013; Affymetrix, Inc., 100, Santa Clara, CA, United States).

We used the Plink software (Version 1.90b3.34; Purcell et al., 2007) for the genome-wide association study. SNPs in autosomal and sex chromosomes were analyzed separately. The quality control parameters of the genotyping results of autosomal chromosomes were as follows: geno 0.1, maf 0.01, hwe 0.000001, and mind 0.1. A Hardy–Weinberg disequilibrium test was not performed for the sex chromosome SNPs. The quality control parameters for the Z and W chromosome SNPs were as follows: geno 0.1, maf 0.1, and mind 0.1. Association analyses were performed with the basic case/control association test of Plink. All *P* values after multiple tests were adjusted by the Bonferroni method with a significance level of *P* < 1.32e-07.

### RNA Sequencing and Differential Expression Analysis

Twelve one-day-old Shouguang chicks from the Experimental Chicken Farm of China Agricultural University were randomly selected, including six EF chicks and six LF chicks. Feather follicles of the wing primary flight feathers and the secondary flight feathers in the left side of each chick were collected and immediately stored in liquid nitrogen for RNA extraction. Total RNA of each sample was isolated using TRIzol^TM^ Reagent (Life Technologies Invitrogen, Carlsbad, CA, United States) according to the supplied manufacturer’s instructions.

The cDNA library construction was performed using the NEBNext^®^ Ultra^TM^ RNA Library Prep Kit for Illumina^®^ (#E7530L,NEB) according to the supplied protocol. A cluster was generated by the HiSeq PE Cluster Kit v4-cBot-HS (Illumia, San Diego, CA, United States) according to the supplied protocol. Pair-end sequencing (PE, 150 bp) proceeded using the Illumia novaseq6000 platform by a commercial service company. Clean reads were generated by removing low-quality bases (Qphred < 20) and raw reads with an adapter pollution or N ratio greater than 5%. Q30 were calculated as the evaluating indicator of clean reads.

Reads mapping and transcript expression level quantification were performed using the workflows of HISAT2, StringTie, and DESeq2 (Pertea et al., 2016). Briefly, paired-end clean reads were aligned against the chicken reference genome (GRCg6a) using the HISAT2 software (Version 2.1.0; Kim et al., 2015). Transcript assembly, GTF document mergence, and transcript abundance estimation were performed using StringTie (Version 2.0; Pertea et al., 2015). The Python script of prepDE.py was used to extract the read count information of each transcript from the coverage values estimated by StringTie^1^. After the results of prepDE.py returned, differential expression analysis was performed using DESeq2 (Version 1.28.1; Love et al., 2014). Our criterion for identifying DEGs was |log2FoldChange| ≥ 1 and *padj* ≤ 0.1.

### Functional Annotation of Differentially Expressed Genes

The functional annotation of differentially expressed genes was performed with DAVID 6.8^2^.

### Quantitative Real-Time PCR

Twelve tissue samples of feather follicles, including six EF chicks and six LF chicks, were collected as described in 2.3 above. Total RNA of each sample was isolated using a RNAprep pure Tissue Kit (cat. #DP431, TIANGEN, Beijing, China). One μg RNA of each sample was used for cDNA synthesis using FastKing-RT SuperMix (cat. #KR118, TIANGEN, Beijing, China) according to the supplied protocol. The primer sequences were designed using Primer Premier 5.0 (Premier Biosoft International, San Francisco, CA, United States) and are listed in Table 1. Quantitative real-time PCR (qRT-PCR) was performed on a CFX96 Real-Time System (Bio-Rad, Hercules, CA, United States) with a 20 μL reaction system using SuperReal PreMix Plus (SYBR Green; cat. #FP205, TIANGEN, Beijing, China) according to the supplied protocol to examine the mRNA expression levels of *PRLR* and *SPEF2*. Each sample had three technical duplicates, and HPRT was set as control. The thermal cycling process was as follows: 95°C for 15 min, followed by 40 cycles of 95°C for 20 s, 56°C for 20 s, and 72°C for 20 s. Relative gene expression was calculated using the 2^–Δ^ ^Δ^ ^*Ct*^ method (Livak and Schmittgen, 2001). We performed the *t*-test method to test the expression differences of the target genes using GraphPad Prism (Version 8; GraphPad Software, La Jolla, CA, United States) and set the significance level at *P* < 0.0001.

**TABLE 1:** The primer sequences for quantitative real-time PCR.

**Primers**	**Sense/antisense**	**Primer sequence (5′–3′)**	**Product size (bp)**
PRLR-F	Sense	TTTTATCCTACCGCCAGTTCC	174
PRLR-R	Antisense	TGATCCTCGCTGTCCTCTACT	
SPEF2-F	Sense	TGGCTACCTCTTTGGAGAACTT	280
SPEF2-R	Antisense	TGCAGGTCTCATTGCTTCCAT	
HPRT-F	Sense	CAACCTTGACTGGAAAGAATGT	171
HPRT-R	Antisense	CAACAAAGTCTGGCCGATAT	

## Results

### Genome-Wide Association Study

**FIGURE 2:**
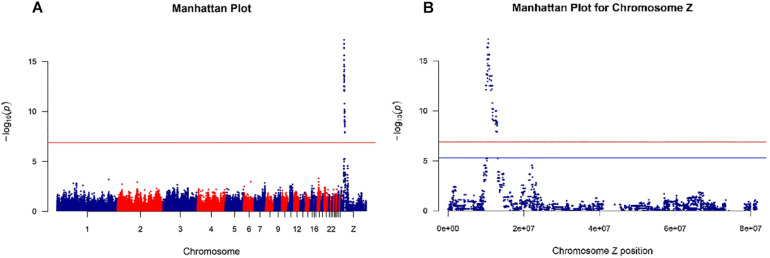
Manhattan plots for genome-wide association study of the feather rate of Shouguang chicks. The red line shows the *P* value threshold of 1.32e-07 adjusted by the Bonferroni method with total SNP numbers after quality control [0.05/(368,657 + 9,207)]. **(A)** Manhattan plot for the genome-wide association study of the feather rate with all SNPs throughout the whole genome. **(B)** Manhattan plot of the Z chromosome for the genome-wide association study of the feather rate. The blue line shows the *P* value threshold of 5.43e-06 adjusted by the Bonferroni method with SNP numbers of chromosome Z (0.05/9207).

The 600K SNP Axiom Genome-Wide Chicken Genotyping Arrays used in this study contained a total of 580,961 SNPs. After quality control, only 368,657 SNPs in autosomal chromosomes and 9,207 SNPs in the Z chromosome were used for the genome-wide association study. All 56 samples from the Shouguang chickens passed the quality control. The Manhattan plots of the GWAS results are shown in Figure 2. From Figure 2 and Supplementary Table 1, we observed that the genomic region of 10.0–13.0 Mb of the Z chromosome was significantly associated with the chick feather rate. There were 371 significant SNPs (*P* < 1.32e-07; Supplementary Tables 1, 2) in the genomic region, and the most important candidate genes for the trait were *PRLR* and *SPEF2* since the SNPs near them had the lowest *P* value and formed a unique peak in the Manhattan plot (Figure 2 and Supplementary Table 2). Among those significant SNPs, genotypes of any one SNP locus were not completely consistent with the phenotype, so we did not think that these SNPs were the causative mutations of the fast or slow feathering phenotype.

The linkage disequilibrium (LD) analysis was also performed on SNPs within the genomic region of 10.0–13.0 Mb of chromosome Z, using Haploview (Version 4.1; Barrett et al., 2005). The results are shown in Supplementary Table 3. From Supplementary Table 3, we observed that all D′ values were greater than 0.69, and most of them were equal to one.

### Overview of the RNA Sequencing Data

A summary of the RNA sequencing data is shown in Supplementary Table 4. A total of 85.01 Gb of raw data were obtained. After filtering, we returned 79.86 Gb of clean data averaging 6.66 Gb per sample and the Q30 value of each sample was greater than 93%. A summary of clean data alignments is displayed in Table 2. The alignment rate of the clean data of each sample was above 93%. These results confirmed that the RNA sequencing data qualified for subsequent analyses.

**TABLE 2:** The summary of clean data alignments.

Sample name*	**Total reads**	**Total aligned reads**	**Multiple aligned reads**	**Uniquely aligned reads**
EF1	23,306,341	93.49%	1,543,196 (6.62%)	19,382,255 (83.16%)
EF2	23,582,797	93.28%	1,528,142 (6.48%)	19,631,660 (83.25%)
EF3	22,418,467	93.29%	889,184 (3.97%)	19,253,151 (85.88%)
EF4	23,686,343	93.64%	1,579,961 (7.04%)	18,580,174 (82.78%)
EF5	22,218,519	93.99%	1,682,564 (7.57%)	18,393,747 (82.79%)
EF6	22,445,556	93.64%	1,579,961 (7.04%)	18,580,174 (82.78%)
LF1	19,598,315	93.33%	1,878,268 (9.58%)	15,595,267 (79.57%)
LF2	22,090,611	93.82%	1,814,998 (8.22%)	18,080,225 (81.85%)
LF3	20,078,650	94.08%	1,517,848 (7.56%)	16,613,145 (82.74%)
LF4	21,962,497	93.78%	1,772,559 (8.07%)	17,940,087 (81.69%)
LF5	23,040,135	93.67%	1,803,841 (7.83%)	18,808,516 (81.63%)
LF6	21,773,897	93.92%	1,480,200 (6.80%)	18,199,899 (83.59%)

*EF1-6, early-feathering chicks; LF1-6, late-feathering chicks.

### Filtering of Differentially Expressed Genes and Functional Enrichment Analysis

**FIGURE 3:**
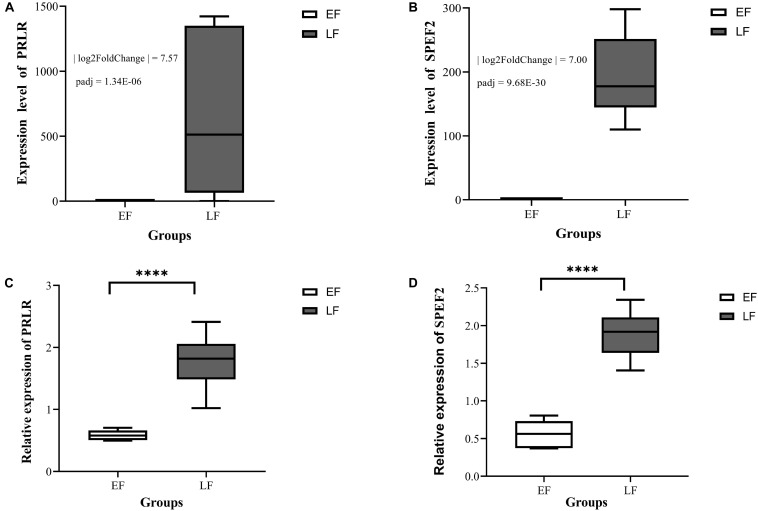
The expression divergences of *SPEF2* **(A,C)** and *PRLR* **(B,D)** between EF and LF Shouguang chicks at one-old age. EF means the early-feathering Shouguang chick group, LF means the late-feathering Shouguang chick group. Panels **(A,B)** were plotted based on RNA sequencing analysis; Panels **(C,D)** were plotted based on the results of quantitative real-time PCR. ****means the values of gene relative expression levels are significantly different between the EF and LF groups at the *P* < 0.0001 level.

After analysis with DESeq2, a total of 501 differentially expressed transcripts (DETs), including 370 DETs of 342 differentially expressed known genes (DEKGs) and 131 DETs of unknown genes (Supplementary Table 5) were found between the EF and LF Shouguang chicks. Among them, 18 DEGs, including 17 DEKGs and one differentially expressed unknown gene (DEUGs), were on the Z chromosome (Supplementary Table 5) and *SPEF2* and *PRLR* were the only two overlapping genes between the results of GWAS and RNA sequencing analysis. The expression divergences of *SPEF2* and *PRLR* between EF and LF chickens were validated by qRT-PCR as shown in Figure 3. From Figure 3, we can see that the expression levels of *SPEF2* and *PRLR* genes in LF individuals were both significantly higher than those in EF individuals.

**FIGURE 4:**
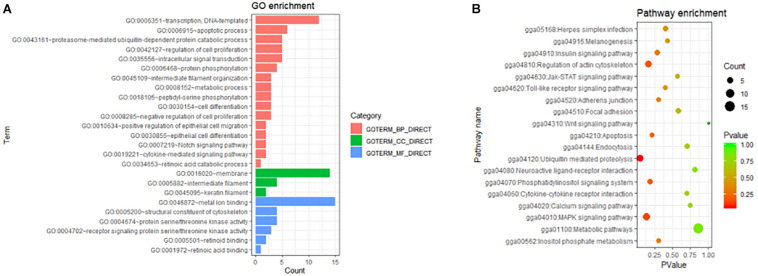
The GO **(A)** and KEGG **(B)** enrichments with differentially expressed genes between early-feathering and late-feathering Shouguang chicks.

All DEGs in the present study may play roles in the phenotype of the chick feather rate. To understand the role of these genes, GO and KEGG analyses were performed with the 342 DEKGs. The results are shown in Figure 4. From Figure 4, we can see that many genes were enriched and were related to cell differentiation and proliferation (GO:0030855, GO:0045605, GO:0030154, and GO:0042127), the keratin filament (GO:0005882, GO:0045109, and GO:0045095), protein serine/threonine kinase activity and peptidyl-serine phosphorylation (GO:0004674, GO:0018105, and GO:0004702), retinoid binding (GO:0005501, GO:0034653, and GO:0048387), ubiquitin mediated proteolysis (gga04120), metabolic pathways (gga01100), the MAPK signaling pathway (gga04010), the JAK-STAT signaling pathway (gga04630), the cytokine-cytokine receptor interaction (gga04060), the wnt signaling pathway (gga04310), and so on.

## Discussion

In the previous reports, *PRLR* was proposed as the late-feathering gene, and the duplication sequence containing part of the *PRLR* gene was regarded as the causative mutation of the LF phenotype in chicken (Elferink et al., 2008; Luo et al., 2012; Bu et al., 2013). However, many Chinese native chickens with the LF phenotype have the duplication sequence, which implies that the duplication sequence can not be the causative mutation of the late-feathering phenotype (Li, 2012; Huang, 2014). Using GWAS, Derks et al. (2018) mapped the feather rate gene of turkey on a segment of the Z chromosome containing 55 protein expression genes, including *PRLR* and *SPEF2*. Based on the transcriptome analyses of chick wing skin tissues, Fang et al. (2018) did not detect a significant expression difference of the *PRLR* gene between the EF and LF chicks, and suggested that *PRLR* only contributed to follicle development, while *SPEF2* was highly related to the growth rate of primary feathers or primary-covert feathers and could be responsible for early and late feather formation. According to the results of GWAS in the present study, *PRLR* and *SPEF2* were two important candidate genes for chicken feather rate. Combined with the results of RNA sequencing analysis, we found that *SPEF2* and *PRLR* were the only two overlapping genes between the results of GWAS and differential expression analysis, which implied that *SPEF2* and *PRLR* were the only two possible candidate genes for the formation of the chicken feathering phenotype in the present study.

The *PRLR* is located on the surface of the target cell membrane and plays a role in many important physiological processes, such as reproduction, metabolism, growth and development, immune regulation, cell proliferation, and differentiation, etc., (Binart et al., 2010). Studies on *PRLR* knockout mice also observed accelerated hair replacement and follicle growth, suggesting that *PRLR* could inhibit hair follicle development (Foitzik et al., 2003; Craven et al., 2006). *SPEF2* plays an important role in sperm differentiation and the formation of sperm tail structure in mice (Sironen et al., 2010). The loss of *SPEF2* function in mice results in spermatogenesis defects and primary ciliary dyskinesia (Sironen et al., 2011). *SPEF2* is also expressed in bone and cartilage, and could regulate osteoblast differentiation and bone growth (Lehti et al., 2018). In a pig study, an L1-insertion within the *SPEF2* gene was associated with litter size in the first parity (Sironen et al., 2012). In chickens, it was also reported that late-feathering cocks had higher semen quality and/or ejaculation quantity than early-feathering cocks (Wei et al., 2005; Cao et al., 2007). These studies above suggest that *SPEF2* is mainly related to spermatogenesis and skeletal development in animals. However, Huson et al. (2014) reported that the gene was also associated with the slick-hair phenotype that was characterized by fine, sleek hair, fewer follicles, and shorter hair length in cattle, which is consistent with our result about the association of *SPEF2* with feather development. So, we inferred that *SPEF2* not only makes a difference in sperm quality between EF and LF chickens but is also involved in the formation of the feather rate phenotype.

In the present study, we obtained 342 DEKGs by RNA sequencing analysis. GO and KEGG analyses with these DEKGs were performed to better understand the molecular mechanism of the feather rate phenotype. Through functional enrichment analysis, we discovered that some genes, including *DNAJB6* [DnaJ (Hsp40) homolog, subfamily B, member 6), *KRT6A* (keratin 6A), *KRT75* (keratin 75), and *LOC431276* (feather keratin Cos1-1/Cos1-3/Cos2-1-like] were enriched in terms of intermediate filament and keratin filament. Beta-keratins play an important role in the morphological and structural diversity of chicken feathers (Ng et al., 2014). A 69 bp in-frame deletion in a conserved region of *KRT75* caused a defective rachis in the frizzle feather chicken (Ng et al., 2012). The absence of the *DNAJB6* co-chaperone prevented proteasome degradation of keratin 18 intermediate filaments in mice (Watson et al., 2007). *KRT6A* might be associated with feather follicle development in chicken embryo fibroblasts (Chen et al., 2017). In mice, the reactivation of *KRT6A* may induce hair follicle development (Gu and Coulombe, 2007). In addition, one of the DEKGs named *ACER1* (alkaline ceramidase 1) also mediated the growth arrest and differentiation of keratinocytes (Mao and Obeid, 2008).

Retinoic acid has an effect on the feather formation and axial orientation of skin appendages (Chuong et al., 1992; Chuong, 1993). In the present study, several GO terms (GO: 0005501, GO: 0034653, and GO: 0048387) related to retinoic acid were enriched, which implies that some genes of the biochemical processes of retinoic acid may play a role in the formation of the feather rate phenotype.

The development of feathers is the result of the proliferation and differentiation of feather follicle stem cells (Yue et al., 2005). Chuong (1993) suggested that the process of cell differentiation and proliferation is related to the morphogenesis of feathers and some anterior hairs. Since several GO terms related to cell differentiation and proliferation (GO: 0030855, GO: 0045605, GO: 0030154, and GO: 0042127) were enriched in the present study, we infer that cell differentiation and proliferation are also involved in the difference of the feather rate phenotype between the EF and LF chicks.

## Conclusion

In the present study, we performed a genome-wide association study and RNA sequencing analyses to detect candidate genes for the feather rate in Shouguang chicken. We found that a genomic region of 10.0–13.0 Mb of the Z chromosome was statistically associated with the feather rate of Shouguang chickens at one-day old. After RNA sequencing analysis, 342 differentially expressed known genes were identified between early-feathering and late-feathering Shouguang chicks. *SPEF2* and *PRLR* were the only two overlapping genes between the results of GWAS and RNA sequencing analysis, which implies that *SPEF2* and *PRLR* are the only two possible candidate genes for the formation of the chicken feathering phenotype.

## Data Availability Statement

The datasets presented in this study can be found in online repositories. The names of the repository/repositories and accession number(s) can be found below: CNGBdb (accession: CNP0001408).

## Ethics Statement

The animal study was reviewed and approved by the Ethics Review Committee for Laboratory Animal Welfare and Animal Experiment of China Agricultural University.

## Author Contributions

HB, JL, and CW conceived and designed the experiments. XL, ZW, and JL performed the field experiments. HB, XL, and ZW analyzed and interpreted the results. XL and HB drafted the manuscript. HB and CW acquired the funding. All authors contributed to the manuscript revision, article, and approved the submitted version.

## Conflict of Interest

The authors declare that the research was conducted in the absence of any commercial or financial relationships that could be construed as a potential conflict of interest.

## Footnotes

1 https://ccb.jhu.edu/software/stringtie/index.shtml?t=manual

2 https://david.ncifcrf.gov/
